# Historical and Chronological Sketch of the Most Important Works on the Dental Art, and the Special Principles Serving as Its Basis, Which Have Been Published from the Days of Hippocrates down to the Present Time. Divided into Three Epochs
*Since the publication of Desirabode's work, many other works have been written upon the diseases of the teeth and mouth.—Eds.


**Published:** 1857-01

**Authors:** 


### ARTICLE III.

*Historical and Chronological Sketch of the most Important Works on the Dental Art, and the Special Principles serving as its Basis, which have been published from the days of Hippocrates down to the present time. Divided into three epochs.** Translated from the French of Desirabode. BY R. A., D. D. S., M. D.

### EPOCH I.—FROM HIPPOCRATES TO THE XVIth CENTURY.

THAT those principles which serve as a basis for the science and art of the dentist cannot be traced back to their origin, is an incontestable fact. In order to dispel any doubts which may arise, in relation to this statement, it will only be necessary to refer to some of the proverbs of Solomon in which certain substances, injurious to the teeth, are formally alluded to; and to remember that HOMER, who wrote two centuries only, subsequent to the time of the son of David, speaks of the functions of the teeth in terms which would do honor to a modern physiologist.

As to the art itself, it is affirmed by HERODOTUS, who lived nearly five hundred years before the Christian era, that, amongst the Egyptians, from the most remote period, a particular caste devoted itself exclusively to its practice. But although we find unmistakable traces of the existence of our art amongst people of the greatest antiquity, we are obliged to acknowledge, with M. Duval, that we are in possession of no valuable knowledge which has been obtained prior to the time of HIPPOCRATES.

This father of medicine had studied the teeth under the three aspects of their anatomy, their physiological history and the treatment of their diseases. That he studied their anatomy is proved by the observation, (which he made in his works entitled, *De dentione, de naturâ hominis*, and *De alimento*,) that

* Since the publication of Desirabode's work, many other works have been written upon the diseases of the teeth and mouth.—EDS.

the germs exist in the fœtus, and also from the name he gave to the last large molar, which he called the wisdom tooth. That he studied them physiologically is proved by this passage from his *Aphorisms:* Cold is injurious to the bones, but heat is very genial to the teeth. Finally that he studied them in relation to their pathology is seen from what he says in his treatise, *De Morbis Vulgaris*, of the diseases of the gums and of the extraction of the teeth. DIOCLES, his disciple, has given the formula of an odontalgic remedy, which however is not found in the only one of his writings which has descended to us—his *Letter to Antigonus*—it was preserved by Galen.

In the following century, that is, about 350 years before the Christian era, ARISTOTLE made his appearance (*Opera omnia*, published at Florence in 1527, and at Paris in 1619;) although what this author said of the teeth is not free from error, he established some facts in relation to their organization. Thus, although he did not dare to separate them completely from the osseous system, he, however, regards them as corresponding to the beak of the bird, and as analogous to the nails and hair.

In the long space of time which elapsed from Aristotle to Galen (about 500 years) the natural history of the teeth seems to have made no advances; but, if we can judge by what has been said by CELSUS, who lived about the middle of this period, of the diseases of the teeth, and the operations performed upon them, we cannot doubt but that considerable attention was paid to their organization. Finally, PLINY, who existed about the time of Celsus, although he has added nothing to what was advanced by Aristotle, speaks of the influence of certain waters upon the teeth. (See the various translations of the 37 books which compose his *Natural History;* amongst others that of C. B. GEROULT, 3 vol. 8vo, published in 1802.)

GALEN, who wrote in 150th year of the Christian era, has given a more exact description of the teeth than any other of the ancient authors; he considered them as true bones—affirmed that they were formed in the fœtus but did not make their appearance through the alveoli till after birth—discovered that they were not devoid of sensibility, and even went so far as to say that the canine were called eye and teeth, because they received branches of the nerve which also supplied the eye, (he, doubtless, intended to designate the anterior dental nerve which traverses the orbit for the purpose of given off branches to the roots of the canine teeth)—finally, he devotes attention to their diseases, and was the first who used the file upon the teeth. (See the Greek Latin edition of his works, published in 1639—1679, by R. Chartier, who has joined them with those of Hippocrates.

Ætius, an Arabian physician, who wrote more than 200 years subsequent to Galen, as he lived in the fourth century, has added to what was left by the latter, some very precise details in relation to the anatomy of the teeth. It was he who discovered the little foramina in the roots which give passage to the nervous filaments. His works were translated from Greek into Latin by Cornarius, in 1567.

Cælius Aurelianus, who, according to Leclerc, lived in the fifth century, has spoken of the teeth; but what we wish to note in relation to this author is, that we are indebted to him for the information that Eristratus, grandson of Aristotle, who flourished about 300 years before the Christian era, was opposed to the extraction of teeth, and, but for him, we would have known nothing of the instruments, deposited in the temple of Delphos, which seemed intended to remind the spectators of the principles of this operation.

Paul of Egina, who wrote in the 7th century, proposed the excision of the teeth when they were adherent to the jaws and when there was any probability of danger in their extraction. (See his Surgical works translated from Greek into Latin in 1593, by Tolet, Lyons.)

Rhazes and Avicenne, Arabian physicians, the true names of whom were, Razi and Abou-ibn-Sina, and who lived, one in the 9th and the other at the end of the 10th century, have spoken of the teeth, without having added any thing of importance to what was left by their predecessors.

But if these various authors have given some attention to dental anatomy and pathology, Albacasis, another Arabian physician, also known by the name of ABOUL-CACEM, and living at the beginning of the 12th century, was the first who gave rules for the replacement of lost teeth, by artificial teeth, either natural or made of bones of the various great mammiferous animals. His works are generally known under the title of the Practical Method, and have many times been translated into Latin. The citations which we have made from Martial who wrote eleven hundred years before him, and those which we made from OVID, CATULLUS, TIBULLUS, PROPERTIUS and JUVENAL prove, nevertheless, that this branch of the art, so important at the present time, was anciently well known. In reflecting upon the luxurious and effeminate life led by the people of Rome and Athens, at certain periods, and the pains they took to procure all the little luxuries, we cannot doubt that this branch of dentistry had attained a high degree of perfection amongst them.

### EPOCH II.—SIXTEENTH AND SEVENTEENTH CENTURIES.

XVI.—In the picture we have sketched of the first works, of which the teeth have been the subject, it will be seen that we have met with nothing written *specially*, in relation to them. All that has been said of them, at least all which has reached us, is, as it were, lost in *general* treatises. It was only in the sixteenth century that works exclusively devoted to an examination of their structure and disease, began to be published.

Among the authors who have the merit of opening the way was URBAIN HEMARD, a surgeon of Lyons, France, erroneously designated Steimard, in the 3d vol. of the large Dictionary of Medical Sciences, (p. 397.) His work appeared in 1582, under the title of: *Recherche de la vraye anatomie des dents, nature et propriété d'icelles, où est amplement discourse de ce qu'elles out de plus que les os, avec les maladies qui leur adviennient.* (Researches upon the true Anatomy of the Teeth, their Nature and Properties; in which is also Amply Treated of, the Differences between them and the bones, with the Diseases to which they are Subject.) If any one will give himself the trouble to even read this little book, which has not been mentioned by any writer upon the teeth, he will really be astonished at the excellent things it contains, and which present: 1st. A faithful exposé and luminous appreciation of the most frequent controverted doctrines of our own day, as, for instance, the question, whether the teeth are true bones; and, if not, in what respect they differ from them; 2ndly. A most rational account of the circumstances under which the structures of the teeth most rapidly undergo alteration; 3dly. Methods of treatment which are undoubtedly, quite as rational as those proposed by most dentists at the present day.

Near the time at which Hemard's work made its appearance in France, four memoirs, also special, were published; two of which appeared in Germany, one in Spain and one in Switzerland. The two former are those of RYFF, G. H., (the same whom Lafaye and Garangeot erroneously named as the discoverers of the circulation of the blood,) and ADAM BODENSTEIN, son of the celebrated theologian of that name, who was the preceptor and friend of Luther. The former of these two memoirs, printed at Wurtzbourg, in 1588, is entitled: *Nützlicher Bericht wie man die Augen Scharfen und die Zähne frisch und fest erhalten-soll.* (Useful Information in relation to the Means of Strengthening the Eyes and of Preserving the Freshness and Solidity of the Teeth;) it is really nothing more than a recapitulation of trite formulas and common place sentences. The second was published at Frankfort, under the title of: *Zahn Arzney*, (Dental Medicine;) it is far from equalling the work of Hermard, which preceded it about six years; it partakes of the eccentric ideas of Paracelsus, whose works the author translated and commented upon.

The two other memoirs here alluded to were written, the former entitled, *Colloquium de Dentione*, by F. M. CASTRILLO, and was published at Madrid, in 1557; the latter, *Disputatio de Dentibus*, by ERASTUS, and was published at Bâle in 1595.

Without wishing to detract from the merits of these various productions, it is, however, important to remark, that they have for the most part, been preceded by the anatomical labors: 1st,

Of VESALIOUS, the founder of human anatomy, who (*De Corporis Humani Fabrica*, Bâle, 1543,) gives a sufficiently accurate description of the external characteristics of the teeth, but who believed, as did also his disciple COLUMBO, (*De Re Anatomicâ*, Paris, 1562,) that the roots of the temporary, serving as germs to the permanent teeth should not be extracted; 2ndly, Of FALLOPICES, (*Observationis Anatomicæ*, Venice, 1561,) who accurately described what is called by modern authors, the *gubernaculum dentis*, which he regarded as a prolongation of the dental follicule of the gum; 3dly, DE COITER VALCHERUS, who first (*De Ossibus et Cartilaginibus Corporis Humani Tabulæ*, Bologna, 1566,) described, with accuracy, the dental pulp and noticed the difference between the ossification of the teeth and the bones; 4thly, Of EUSTACHIUS, the object of whose researches, (see in his small anatomical treatises the chapter *De Dentibus*, Venice, 1574,) was the discovery of the germs of the canine and incisor teeth, thus overturning the opinion of Vesalius, who engaged in long discussions to demonstrate that fractures of the teeth are never repaired.

From other works, specially devoted to the teeth, are known to have been published in this century; the first was by JEAN BENEDICTUS, a German physician, who in 1552, reported cases of palatine teeth; the second by PIERRE MONAVIUS, published under the title of *De Dentius Affectibus*: Basil, 1578. The authors of the two others are HORSTIUS and INGOLSTETTER, who wrote in 1595, a description of a tooth of gold, and with which a young child of Silesia was said to have been born. Finally, amongst authors, who in their works on general subjects, treated of the teeth, we should cite AMBROSE PARE, who (in his complete works, published in 1582) gave a very exact description of these organs, spoke of the transplantation of teeth, of artificial teeth, and described several palatine obturators of very ingenious construction, to which subject, an author named A. PETRONIUS, (*De Morbo Gallico*, 1563,) had also called attention; INGRASSIAS, J. P., who in his commentaries upon the work of GALEN, (*De Ossibus*, Venice, 1568,) also describes the successive changes which the teeth undergo in the process of development; MERCURIAL JER. called MERCURIALIS, who contended, about 1580, that, as nature never intended to produce disease, dentition could not be regarded as a disease; PLATER, (*De Corporis Humani Structurâ;* Bâle, 1583,) who regarded the teeth as entirely inorganic, and consequently incapable of any other kind of sensation than that received from the gum surrounding them; and TRASTUS, TH., who in a series of letters, published in 1595, devoted one to the teeth, under the title: *Disputatio de Dentibus.*

XVII.—The number of works upon the teeth, necessarily augment in proportion as letters are more cultivated and as the sciences advance. The seventeenth century produced a greater number, independently of what was said upon the subject in general treatises upon anatomy by the following named authors: THOMAS BARTHOLIN, (*Anatomica Bartholini,* 1641;)—HYGHMORE, NATH., (*Corporis Humani Disquisit. Anat.;* La Heaye, 1651,) who first described the maxillary sinus, which still bears his name;—DU BOIS or DEL BOE, commonly called SYLVIUS, (*Opera Omnia;* Amsterdam, 1672;)—DUVERNEY, J. G., who in an excellent description of the teeth, (published in 1683,) observed that in their development and nurture, they bear a strong analogy to feathers, to the nails, to horn and hair;—DIEMERBRŒCK, ISB., (*Opera Omnia Anatomica et Medica;* Utrecht, 1685,) who reports many cases of very remarkable dental anomalies, but who commits the fault, unpardonable for the period at which he lived, of maintaining that the teeth are formed after birth, and that the roots of the temporary, give origin to the permanent teeth;—ABEILLE SCIPIO, (*Historie des os,* or *Anatomie de la tête et de ses parties;* Paris, 1685; "History of the bones, or Anatomy of the head and its parts;") —GAGLIARDI, DOM., (*Anatome Ossium Novis Inventis Illustrata;* Rome, 1689,) the first who described the enamel of the teeth, and obtained sparks from it, by striking it against the steel;—HAVERS CLOPTON, to whom was attributed the discovery of the synovial glands; who (Traité d'ostéologie, 1691, "Treatise upon Osteology") established clearly, the difference between the enamel and dental bone, and asserted, that in the latter he had discovered traces of filaments of nerves ;—and PH. VERHEYEN, (*Corporis Humani Anatomia*, etc., *cum tabulis*, 1693,) whose belief in a perfect analogy between the teeth and nails, was so great as to lead him to the conclusion, that like the latter, they continued to grow without cessation during life.

The special works published in the XVII century, independently of the general treatises of which we have spoken, are as follows, in the order of their publication:

1602. FOREEST, *De Ægritudinibus gengivarum Dentium, etc.;* Leyde. HEURNIUS, *Tractatus de Morbis Oculorum, Aurium et Dentium;* Leyd.

1620. VIGIER, *Tractatus de Catharrhò, Rhumatisme Vitüs Dentium*; Geneva.

1622. GILLES, ARNAUD, *Le fleur des remèdes Contie le mal de dents ;* Paris. (The Cream of Remedy for toothache.)

1626. LIDDELINS, *Tràctatus de Dente Aureo ;* Hamburg.

1627. TEXIER, JEAN, *Ergō quibus Rariores Dentes,* Βραχυβιωτεροι, 4to.; Paris.

1629. SENNERTUS, *Dissertatio de Dentium Dolore ;* Witeb.

1630. STROBELBERGER, J. STEPH. *Tractatus de Dentium Podagrâ Sive de Odontalgiâ in quo Dentium Sine et Cum Ferro Artificiosè extrahendorum Varii Modi Theoricè et Practicè Proponuntien, etc.;* Lips.

1631. ZEIDLER, *Dissertatio de Dolore Dentium ;* Leps.

1633. DUPONT, *Remède Contre le mal de dent*, (Remedy for Toothache,) this consists in extracting the diseased tooth, and substituting for it another, taken at the choice of the patient, so says the author, from a dead or living subject.

1639. LOESEL, JEAN, *Dissertatio de Dolore Dentium.*—PAULI, *Dissertatio de Dolore Dentium ;* Hafni.

1642. PALLU, *An Dentium Dolorem Conferat Tabacum.*

1645. SCARDOVI, *Dissertatio de Dentibus ;* Argent.

1651. STRASBURG, Dissertation upon Odontalgia, (in the Greek language;) Reg.

1656. LAMBERT, *Commentaires Sur la Carie et Corruption des os.* (Commentaries upon Caries and Putrefaction of the Bones, 8vo. Marseil.

1660. BAUHENIUS, *Dissertatio de Odontalgiâ.*

1661. MOEBIUS, *De Odontalgiâ, Seu de Dentium Statu Naturali atque Prœternaturali;* 4to. Jena.

1663. AURENIUS, *Dissertatio de Catarrho et ex lo Descendentibus, Odontalgiâ,* etc.; Rostoch.

1664. SEBIZIUS, *Disputationes quatuor de Dentibus;* Stras. The same author, *De Dentibus, Urinâ et Morbis Contagionis,* etc.; Argent.

1669. ROLFINCK, *Dissertatio de Odontalgiâ;* Jena.

1672. CONRINGIUS, *Dissertatio de Naturâ et Dolore Dentium.*—HEYE, *Dissertatio de Dolore Dentium;* Helms.

1675. STISSLER, *Dissertatio de Odontalgiâ;* Lugd.

1678. LEICHNER, RICHARD, *Dissertatio de Atrocessimo et tantùm non Intolerabili Dentium Dolore;* Erfort.—WEDEL, *Dissertatio de Dentitione Infantium;* Jen.

1679. MARTIN, *Dissertation sur les dents;* (Dissertation upon the teeth;) Paris.

1681. CRAUSIUS, *Dissertatio de Odontalgiâ;* Jena.—TYSEN, Observations on the hair, teeth, and bones found in different parts of the body.

1682. FLEURIMON, *Moyens de Conserver les dents bellcs et bonnes;* (Means of preserving the teeth in fine and good condition;) Paris.

1683. VATER. *Dissertatio de Odontalgiâ;* Witeb.

1688. TRECURTH, *Dissertatio de Dentitione;* Hal.—STREITLEIN, *Dissert. de Dentitione;* Altd.—GOECKEL, EBERB. *Epitome Theoriœ et Practicœ de Odontalgiâ;* Nordl.

1692. FRANK, *Dissertatio de Odontalgiâ;* Jena.—BROUWER, *Dissertatio de Odontalgiâ;* Eyd.

1693. MYRRHEN, *Dissertatio de Odontalgiâ;* Giess.

1694. ORTLOB, *Dissertatio de Dentione Puerorem Difficili;* Lieps.—RAU, *Dissertatio de Ortu et Generatione Dentium;* Lugd.

1695. PLANER, *Dissertatio de Odontalgiâ;* Tul.—ZIEGLER ADRIEN, *Dissertatio de Odontalgiâ;* Uth.

1697. BRENDEL, *Dissertatio de Odontalgiâ.*—VESTI, *Dissertatio de Odontalgiâ;* Erf.

1698. HOFFMAN, FREDERICK, *De Dentibus Eorumque Morbis et Curâ;* in 4to., Hal. This paper is printed in his complete works.

1699. PESTORF, *Dissert. de Dentitione Difficili;* Ultr. In the memoirs of the Academy of Sciences of this year, we find a dissertation by LAHIRE, on the structure of the teeth.

1700. HOFFMAN, (before cited,) *De Remediis Antodontalgicis;* Hal.—HESLOP, *Dissertatio de Dentitione Infantium Difficile et Laboriosâ;* Leid.—L. SCHELHAMMER, *Dissertatio de Odontalgiâ Jactu Sanandâ;* Kiel, and in 1711 at Jena.

### EPOCH III.—EIGHTEENTH AND NINETEENTH CENTURIES.

We have necessarily reached that period which has been most fertile in the production of works upon the teeth; and it is fortunate that in proportion to their number is their increase in importance. However, one-half, at least, of those we shall mention, seem rather to have been the result of that species of *Cacöethes Scribendi,* which seems to have seized all the minds of the otherwise brilliant court of the eighteenth century, and to have been dictated rather by a desire to attract the attention of the multitude than to add anything toward the real advancement of the art. Without contradiction, the most remarkable of all which appeared in this century is that of PIERRE FAUCHARD, whose doctrines have served as a basis for all that has been done and written since his time; we have, therefore, made it a duty in the course of this treatise, as often as occasion presented itself to raise him from that oblivion, in which his contemporaries and some practitioners of our epoch, would fain have seen him buried.

Whatever may be their merits, the treatises which appeared in the eighteenth century, are as follows:

1704. CRAUSE, *Dissertatio de Dentium Sensu;* Jena.

1706. *Secrets nouveau et expérimentés pour Conserver la beauté des dents,* (New and tried Secrets for the Preservation of the Beauty of the Teeth:) 12mo. Paris.

1707. PACHEUS, *Dissertatio de Dentium Dolore:* Basil.—

DIONIS PIERRE, in his Treatise on Operations published in this year describes the principal persons who operate upon the teeth, exposes and contemns the charlatanism of the dentists of his epoch.

1708. LUDOLF, *Disputatio de Morbis Gengevarum;* Erf.

1711. HEISTER, *Dissertatio de Dentium Dolore;* Altd.

1714. HOFFMAN, (before cited,) *Dissertatio de Dentibus eorum Morbus et Curâ:* Halæ.

1715. PETIT, J. L., *De quelques unes des functions de la bouche,* (of some of the functions of the mouth:) Paris.

1716. CUMME, A. CH. GEORG., *Dissertatio de Dentium Historiâ Physiologicè Pathologicè et Therapeuticè Pertractâ:* Helmst.

1717. CRON, LUDW., *Der beim Aderlassen und Zahnausziehen gesheckte Barbier Gesell,* (The Journeyman Barber Skillful in Bleeding and Tooth-drawing:) Leips. 8vo.

1718. EHINGER, *Dissertatio de Odontalgiă:* Altd.

1720. BEURLIN R. PHIL., *Dissert. de Dentitione difficili:* Altd.—DESPRE, *Dissert. de Dentitione difficili:* Erf.

1723. GARENGEOT, *Nouveau traité des instruments de chirurgie,* (New Treatise upon Surgical Instruments:) 2. vol. 12mo. Paris.

1727. VALENTINI, *Dissertatio de Vascillatione, Casu et Reparatione Dentium:* Giess.

1728. LOESCHER, *Dissertatio de Dentibus Sapientiæ, eorumque Morbis:* Wittemb.—FAUCHARD, PIERRE, *Le Chirurgeon-dentiste, ou Traité desdents,* (The Surgeon Dentist, or a Treatise on the Teeth:) Paris, 2 vol. 12mo, with 42 plates. There are two other editions of this work, one published in 1746, and the other in 1786.—SCHWARDT, JEAN-CHRIS., *De Dentibus Sapientiæ eorumdemque Morbis:* 4to, Wittemb.

1732. JETUZE, J., *Difficile Infantum Dentitione:* Erfor.—KRAUTERMANN, VAL., *Sicherer Augen und Zahnarzt,* (The Safe Oculist and Dentist:) 8vo. Arnstadt.

1733. KUCHLER, *Dissertatio de Ulceribus Dentium Fistulosis:* Leips.—BRACHMAEND, *De Ulceribus Dentium Fistulosis:* Leips.

1734. Diest, Jean de, An hæmorrhagia ex Dentium Evulsione Chirurgici Incuriâ lethalis? Affirm Questio Medica-Chirurgica: 4to, Paris.

1735. Vase, Ergo Hæmorrhagia ex Dentium Evulsione Chirurgici Incuriâ lethalis: Paris.

1737. Alberti Mich., Dissertatio de Dentibus Serontinis Sapientiæ Vulgo Dictis: Halæ. Gerauldy, l'Art de Conserver les dents, (The Art of Preserving the Teeth:) Paris. Diechmann, Sur les Dents de Sagesse (On the Wisdom Teeth,) an inaugural thesis.

1738. Hebenstreist, J. E., De Dentitione Secundâ Juniorum: 4to, Leips. Zbonatriet, De Dentitione Secundâ Juniorum: 4to, Leips.

1740. Junker, J., Dissertatio de Dentium Affectibus: Halæ. Kemme, Dissertatio Sistens Dentium Historiam, Physiologicé Pathologicé et Therapeuticè Pertractatam: Helms. Lavani, Joseph, Trattaro Sopra la qualità de denti, Col modo di Cavarli, etc. (A Treatise on the Nature of the Teeth and the Methods of Extracting them:) 4to, Florenza. Mongin, Ergo pregnanti Mulieri acutissimo Dentis Dolore Laboranti ejusdem Evulsio.

1741. Bunon Robert, Dissert sur un préjugé concernant les maux de dents des femmes grosses (Dissertation on a prejudice concerning tooth-ache of pregnant females:) Paris.

1742. Hoerlock Jos., Practical Treatise on Dentition: London. Hurlock, Same title. (This, doubtless, is the same work with the author's name altered.)

1743. Bunon, (already cited,) Essai sur les maladies des dents où l'on propose de leur procurer une bonne conformation dès la plus tendre enfance et d'en assurer la conservation. (Essay on the Diseases of the Teeth, in which it is proposed to obtain their good conformation from the earliest infancy, and to insure their preservation:) in 12, Paris.

1744. Ferrein, Deux Memoires lus à l'Academie des Sciences sur les Mouvements des deux mâchoires. (Two papers on the Movements of the Two Jaws, read before the Academy of Sciences.)

1745. JUNKER, (before cited,) Dissertatio de Dentitione Difficili: Halæ. LAUBMEYER, Dissert de Dentibus: Reg.

1746. HEISTER, (before cited,) Epistola de Pilis, Ossibus et Dentibus in variis Corporis Humani Partibus Repertis: Helms. JUNKER, (before cited,) Dissertatio de quatuor Præcipius Infantum Morbis. BUNON, (before cited,) Experiences et demonst., faites, etc., pour servir de suite et de preures à l'Essai sur les maladies des dents (Experiments and Demonstrations made for the Purpose of a Continuation and Proof of the Essay on the Diseases of the Teeth.)

1747. GUNZ, J. G., Programma de Maxillæ Articulo et Motu: Leips.

1748. HILSCHER, Dissertatio de Odontalgiâ: Jenæ. PRYFF, Nützlicher Bericht wie man die Augen Scharfen und die Zähne frisch und fest erhalten soll. (Useful Information in Relation to the Means of Stregthening the Eyes and Preserving the Teeth, fresh and sound.) Wurb. in 12.

1749. HERISSANT, F. DAV., Recherches sur les usages d'un grand nombre de dents du Camix Calcarios. (Researches on the use of a great number of the teeth the Camix Calcarios.)

1750. LECLUSE N., Traité utile au public, où l'on enseigne la méthode de methode de rémedier aux douleurs et aux accidents qui précèden et accompagnent la sortie des premières dents, etc., (A treatise useful to the public, in which is taught the methods of remedying the pains and disorders which precede and accompany the eruption of the first teeth:) Nancy.

1751. APIUS, De Dentium Præsertim Infantium Difficili: Ellang. JANKE, Dissertatio de Dentibus Evellendis, and also, De Ossibus Mandibularum Puerorum Septiennium; Leips. SCHMIEDEL, Dissertatio de Dentitione, Præsertim Infantum Difficili; Erf. SCHŒFFER, I. CRET., Die Eingebildeten Würmen in Zähnen, etc. (Imaginary Worms in the Teeth;) Ratisb. in 12.

1752. BUECHNER, AND. ELI.,Dissertatio de Curâ Dentium ad Sanitatem Proficuâ; Halæ. TOLVER, A., A Treatise on the Teeth; London.

1753. Gunz, (before cited,) Programma de Ozenâ Maxillarum ac Dentium Ulcere; Leips. Le Monnier, Dissertation sur les maladies des dents, (Dissertation on the Diseases of the Teeth.) Ludwig, Programma de Cortice Dentium; Leips.

1754. Lecluse (before cited,) Nouveaux Elements d'Odontologie, (New Elements of Odontology;) Paris. Bourdet, Lettre á M. D., (Letter to M. D.,) The same author, Eclaircissements an sujet de cette lettre, (Explanations in connection with this letter.) Brouzet, Essai sur l'éducation médicale des enfants et leurs maladies, (Essay on the Medical Training of Children and their Diseases;) Paris, 2 vols. 12mo.

1755. Lecluse, (before cited,) Eclaircissement pour parvenir á préserver les dents de la carie, etc., (Explanations having for their object the preservation of teeth from decay;) Paris. Winslow, Remarques sur le Memoire de M. Ferrein, concernant le mouvement de la machoire in ferieure, (Remarks on the Essay of M. Ferrein in Relation to the Movements of the Lower Jaw,) Paris, 4to.

1756. Jourdain, Traité des maladies et des opérations réellement chirurgicales de la bouche et des parties qui y Correspondent; Suivi de notes, observ., consult, interessant., taut ancien. que moder. (Treatise on the Diseases and Operations, Truly Surgical, of the Mouth and of the Corresponding Parts: followed by notes, observ., consult., both ancient and modern.) Paris, 2 vol. 8vo.—The same author, Nouveaux Elements d'odontologie; (New Elements of Odontology;) Paris. Lentin, Leb. Benj., Bemerkungen von der weikung der Electrischen Erschütterung in Zahnweh. (Remarks upon the Action of the Electrical commotion in tooth-ache. Philip, Pfaff, Abhandlung von den Zähnen des mensclichen Körpers und von ihren kraukheiten; (Treatise on the Human Teeth and their Diseases;) Berlin.

1757. Aurivillius, Dissertatio de Dentitione Difficili; Ups. Bourdet, (before cited,) Recherches et observations Sur toutes les parties de l'art du dentiste; (Researches and Observations on all the Branches of the Dental Art;) Paris, 2 vol. 12. Vandermonde, C. Aug., Ergo Infantum a Dentitione Convulsionibus vel Soporibus Repetitus Catarthi Corum Usus; Paris, 4to.

1758. Herrisant, (before cited,) Nouvel. Recher Sur la Conformation de l'émail des dents et celle des gencives. (New Inquiries into the Conformation of the enamel, and of the gums.) Junker, (already cited,) Dissertatio de Odontalgiâ; Halæ.

1759. Duchemin, Sur la Carie des dents le lait; (Upon Caries of the Temporary Teeth;) Journal de Trevoux, Fevrier. Bourdet, (before cited,) Soins faciles pour la propeté de la bouche et la conservation des dents; (Easy means of keeping the Mouth in good Condition, and of Preserving the Teeth;) Paris.

1760. Jourdain, (before cited,) Traité des dépôts dans les sinus maxillaires des fractures et de caries, Suivi de reflexions sur toutes les operations de l'art du dentiste; (Treatise on Depositions in the Maxillary Sinuses, of Fractures and Caries; to which is appended Reflections on all the Operations of the Dental Art;) Paris. Beaupreau, Claude, G., De Dentibus, Theses Anatomica Chirurgicæ; Paris.

1761. Drouin, Sur les maladies des dents; (On the Diseases of the Teeth;) Strasb.

1762. Beaupreau, (before cited,) Dissertation Sur la propreté et la Conservation des dents;) Dissertation on the cleanliness and preservation of the Teeth;) Paris. The same, Journal de Mèdecine, tome xxi. Bourdet, (before cited,) Dissertation Sur la propreté et Conservation des dents; (Dissertation on the Cleanliness and Preservation of the Teeth;) Paris, in 8vo. The same author, Dissertation Sur les dèpôts du sinus maxillaire. (Dissertation on Depositions in the Maxillary Sinus.) Vacher, Dissertatio de Dentium Accidentibus, Paris; there is one edition of 1767.

1766. Glaubrecht, Dissertation de Odontalgiâ; Argento. Jourdain, Essai Sur la formation des dents, Comparée avec celles des os, Suivi de pluissieurs expériences taut Sur les os que sur les parties qui entrent dans leur Composition; (Essay on the Formation of the Teeth Compared with that of the

Bones; followed by Many Experiments on the Bones, and also on the Parts which enter into their Composition;) 1 vol., 12mo. Paris. BRUNNER, J. B., Einleitung zu den Wissenschaften eines Zahnartzes; (Introduction to the Art of Dental Surgery;) Vienna or Leipsic, 8vo. YSABEAU, M., a Surgeon of Gien, in the twenty-fifth volume of the Journal of Medicine, published in this year, has presented cases of teeth, which made their appearance at the ages of 80, 92 and 120.

1767. DISDIER, Histoire exacte des os; (Exact History of the Bones;) Paris. PASCH, J. G., Abhaudlung von den Zähnen, des Zahnfleisch, der Kiefer, Kraukheiten und heid art; (Treatise on the Teeth, the Gums, the Jaws, and their Diseases;) Vienna. BRUN, ANT., De Dentitionis Accidentibus; Paris, 4to.

1768. RUFLINI, B., A Treatise on the Teeth, their structure and various diseases, etc; London, 8vo. GRACBNER, C. A., Gedanken über das Hervor Rommen und wechseln der Zähne. (Reflections on the Eruption and Shedding of the Teeth.) LEROY DE LA FAUDIGUERE, Manière de prevenir et de guérir les maladies des gencives et des dents; (Method of Preventing and Curing Diseases of the Gums and Teeth;) Paris, 12mo.

1769. BEAUPREAU, (before cited,) Lettres à M. Cauchois sur les maladies du sinus maxillaire; (Letters to M. Cauchois, on the Diseases of the Maxillary Sinus;) Paris. CURTIS, a Treatise on the structure and formation of the Teeth; London. TIMÆUS, a treatise on the tooth-ache; 8vo. London. RAULIN, De la Conservation des dents des enfants; (On the Preservation of the Teeth of Children;) Paris, 2 vol. 8vo. VAN SWIETEN, Traité des maladies des enfants; (A treatise on the Diseases of Children;) 1 vol. 12mo.

1770. BERDMORE, a Treatise on the Disorders and Deformities of the Teeth and Gums, illustrated with Cases and Experiments; London. ŒETINGER, Dissertatio de Ortu Dentium, etc; Tub. KOBER, J. JAC., Anatomiæ Comparatæ Specimen Osteologicum de Dentibus; 4to., cum figuris; Basil.

1771. HUNTER, Natural History of the Teeth and their Diseases; London. Translated into French in 1773, and into

German in 1780, and again recently (1841) into French by M. Richelot; P. BODDAERT, in 1781, made an excellent Latin and Dutch translation of this remarkable work. BRUNNER, AD. ANTOINE, Abhandlung von der hervorbrechung der Melchzaehne; (Treatise on the Eruption of the Milk-teeth;) A. D. B., XVI., B., p. 619. WEYLAND, F. S., Disputatio de Ozenâ Maxillairi Cum Ulcera Fistuloso ad Augulum Oculi Intemum Complicato; Argento.

1772. ELOY, Dissertatio de Remedüs Anti Odontalgicis; Vien. LEWIS, M., An essay on the Formation of the Teeth, with a supplement containing the means of preserving them; 8vo. London. AUZEBY, PIERRE, Traité d'odontalgie ou description des maladies qui affectent la bouche, et des moyens de les guerir; (A treatise on Odontalgia, or a Description of the Diseases which affect the Mouth, and the means of curing them;) Lyon, in 12. DEFRITSCH, Dissertatio de Dentibus; Vienna. SHEERS, Dissertatio de Dentibus; Traject.

1775. BURNET. A Dissertation on the Teeth and Gums: London. COURTOIS HON. GAIL., le Dentiste, Observateur, ou recueil d'observations tant sur les maladies qui altaguent les gencives et les dents que sur les moyens de les guerir. (The Observing Dentist, or a Collection of Observations on the Diseases which attack the Gums and Teeth and also upon the Means of Curing them.) Paris in 12. FOUCHON, Propositiones de Dentium Vitüs: Paris.

1776. HEESCHER, (before cited,) Remarques sur les dents, fondées sur la practique. (Remarks upon the Teeth founded upon Practice:) Jeno. POLH, PR., De Difficili Infantum Dentitione: Leips.

1778. COURTOIS, (before cited,) Sur l'etat et les maladies des dents. (On the Condition and Diseases of the Teeth.) Gotha. JACKSON SEG. HEN., Dissertatio de Physiologiâ et Pathologiâ Dentium: Edinb. MEYER, J., Abhandlung von den gewöhulichen zahn Krank heiten. (A Treatise on the most Common of the Diseases of the Teeth:) Hanau. PLENCK, JO. JACQ., Doctrina de Morbus Dentium et Gingivarum: Vienna, 8vo.

1779. BENNET, A Dissertation on the Teeth: London. HEB-

ERT, Le Citoyen Dentiste, (The Citizen Dentist:) Lyons, 12. BROUSONNET, P. M. ANG., Consider. Sur les dents in gener et sur les organ, qui en tiennent lien. (Considerations upon the Teeth in General and upon the Organs by which they are produced.) A paper read, in this year, before the Academy of Sciences, and containing a Comparison of the Human Teeth with those of quadrupeds. It is inserted in [the vol. of Memoirs of the Academy of Sciences for 1787.

1780. KRANSE, R. W., De Odontalgiâ: Jena. MURRAY, Programma de Dentium et Pilorum in Ovario Generatione: Upsal. Prochaska De Decremento Dentium Corporis Humani, Accedit Causarum Dentitionis Secundæ Elucedatio: Vienna.

1781. COLONDRE, Essai sur les plus fréquentes maladies des dents, etc. (Essay on the most common Diseases of the Teeth:) Geneva. ORLOVIUS, Dissertatio de Hemorrhagiâ Oris: König. PLISSON, Observations sur une maladie extraordinaire des gencives. (Observations on an Extraordinary Affection of the Gums:) Lyons.

1782. BUECKING, Vollständige Anweisung, Zum Zahnausgiehen. (Complete Instructions for the Extraction of the Teeth:) Steudal. VAN-DEN-BELEN, Dissertatio de Odontalgiâ: Lava.

1784. LE MONNIER, (before cited,) Lettre a M. Mouton. (Letter to M. Mouton:) 8vo. ANDREE, Dissertatio de Odontagris ad Dentes Evellendos Necessarüs eorum vi Mechanicâ et Applicatione: 4to. Leips. LEMAITRE, Rapport fait à la Société des inventions et decouvertes sur les denticrs serfectionées. (Report made to the Society of Inventions and Discoveries in relation to improved sets of Artificial Teeth:) Paris.

1786. GENLIS, J. C., Progr. Observ. de Dentitione tertiâ. MOUTON, Essai d'odontotechnie ou dissert, sur les dents artificielles. (An Essay on Odontotechny or Dissertation on Artificial Teeth:) Paris, 12. BOTOT, Le Cherurgien Dentiste. (The Surgeon Dentist:) Paris, 12. UNDERWOOD, Traité des malad. des en fants. (A Treatise on the Diseases of Children,) translated from the English by LEFEVRE DE VILLEBRUNE. GEHLER, J. SAM. TRANG., De Dentitione Citeâ: Leips., 4to.

1787. AUVITY, JEAN, Mémoire sur les maladies aphthenses des nouv.-nés. (A Paper on the *aphthous* Disease of newly-born Infants:) Société roy. de Méd., tome 9.

1788. ALLVEY, Dissertatio de Dentitione Morbisque ex eâ Pendentibus: Edinb. KULENKAMP, Dissertatio de Difficili Infantum Dentitione: Harderow. LENIA, Plusieurs Observations Sur un nouveau moyen de guérir Certaines donleurs des dents. (Several Observations on a New Method of Curing certain pains to which the Teeth are subject:) Lyons. WOOFFENDALE, Practical Observations on the Human Teeth, 8vo, London.

1789. CAMPANI, A., Odontalgia, Ossia Traltaro Sopra i denti. (Odontalgia or a Treatise upon the Teeth.) The same author, De Denti e loro Cura, e la maniera di estrarti. (Of the Teeth, their Preservation, and Method of Extracting them:) Fiorenza. DUBOIS DE CHEMANT, Dessèrt. Sur les avantages des nouval. dents et ratêliers artificiels, etc. (Dissertation on the Advantages of the New Teeth and entire artificial sets:) Paris. BOTOT, (already cited,) Avis au peuple Sur les Soins necessaires pour la propreté de la bouche. (Advice upon the care Necessary to keep the Mouth in a Cleanly Condition:) Paris, 12mo.

1790. Lettre sur les dents artificelles. (Letter on Artificial Teeth:) Paris. GIREAU, J., Die gute Mutter, oder Abhandlung von den Mitteln Seinen Kindern einen Starken, dauerhaften Rœrpen, besonders den glucklichen Zähnen zu verschaffen. (The Good Mother, or a Treatise on the means of Procuring for Children good and durable health and especially Sound Teeth:) Brannschw., 8vo, A. D. B. iii, B., p. 411. KRAUSE, C., Dissertatio de Primâ Puerorum Dentitione: Francf. POSEWITZ, Semeilogia aphtharum Idiopaticarum et Symptomaticarum: Vetrib.

1792. MEKEL, Dissertatio au Morbi qui Dentium translat. Sequunt. veneres Lint necne? Hal. PINEL, (The celebrated individual of that name.) Mechanism de la luxation de la Machoire; Medeciné eclairée; tome 3, p. 183. (Mechanism of the luxation of the Jaw; The Enlightened Physician, vol. 3, p. 183.)

1793. KŒNEN, Dissert. de Precipuis Dentium Morbis: Frank. BRING, Observ. in Hodiernam de Dentibus precipue Hominum Doctrinam: Lundæ. RICCI, Principes d'Odontotechnie ou reflexions sur la Conservation des dents et des gencives. (Principles of Odontotechny or Reflections on the Preservation of the Teeth and Gums:) Paris, 8vo. WALKEY, On the Diseases of the Teeth, their Origin Explained, &c.: London.

1794. PLOUGUET, G. G., Primæ Lineæ Odontidis, Sive Inflammationis ipsiorum Dentium, Dissertatio: Tub.

1795. GRUN, Dissertatio de Odontalgiâ: Jena.

1796. HIRSCH, FREDERIC, Praktische Bemerkungen, etc. (Practical Remarks, &c.:) 8vo, Jena. CAILLEAU, J. M., Avis aux mères de famille, etc. (Advice to Mothers of Families, &c.:) Bord. GESCHENK, Zur Personen beidenlei Geschechts die Zähne geschmedmed Schön Zu herhaten. (Advice to Persons of Both Sexes upon the Manner of Preserving their Teeth in Fine Condition:) Frankfort. TENON, Mémorie Sur une méthode particulière d'etudier l'anatomie, employée, par forme d'essai, à des recherches Sur les dents, et les os des mâchoires. (Paper on a Peculiar Method of Studying Anatomy applied, in the form of an Essay, to researches on the Teeth and Bones of the Jaws;) inserted in the first volume of the "Memoires de l'Institut," printed in this year. The same author, Second essay d'etudes par époques, des dents molaires, etc. (Second essay on the Study by Epochs of the molar Teeth, &c.:) same vol.

1798. BLACKE, Dissert. de Dentium Formatione et Structurâ in Hominibus et Varüs Animalibus: Edinb. MAHON, le Dentiste-Observateur. (The Observing Dentist:) Paris, 12. WAGNER CONRAD BERNARD, Dissertatio de Dentitione Difficili a Dubüs C. L. Wichman Vindicatæ: 4to, Jenæ.

1799. BLUMENTHAL, C. A., Nähere Srüfung der Ætiology der Zahnarbeit, etc. (Closer Examination of the Etiology of Dental Surgery:) Steudal, 8vo. NICOLAI, De Varüs Dentium Affectibus, eorumque in Sanitate Influxu Dissert.: in 4to, Jenæ. PALDAMUS, Dissertatio de Dentium Morbis: Hal. DESESSATS, Education Corporelle des Enfants. (Physical Education of Children.)

1800. LUDWIG, (before cited,) Dissertatio de Dentitione Difficili: Leips. FICHER, Sur les divers. form. des os de la mâch. dans plus. espèc. d'animaux. (On the varieties of form of the Bones of the Jaw in Various Species of Animals:) Leips. KREBEL, J. L., Dissertatio Inauguralis de Dentitione Diffici.: Leips.

1801. CAILLEAU, (already cited,) Deux Memoires sur la dentition. (Two Papers on Dentition:) Bord., 8vo. HILSCHER, Second edition of the work published in 1776. SCHMIDT, Kunst Schöne Zähne von Jugend auf zu erhalten. (Art of Keeping the Teeth fine from youth up:) Gotha. The same author, Quelq. mots à ceux qui desirent maintenir leurs dents dans un bel état. (Some Words to those who Desire to preserve their Teeth in Fine Condition.) SKINNER, A Treatise on the Human Teeth, concisely Explaining their Structure, &c.: New York.

1802. ARNEMANN, Système de Chirurgie; le troiseme paragraphe du Traité des maladies des dents. (A System of Surgery; the third paragraph of the Treatise upon Diseases of the Teeth:) Gœtt. BOTOT, (before cited,) Moyens pour Conserver les dents. (Means of Preserving the Teeth.) CAIGNE, FR., Sur la Dentition des enfants du Premier âge, et les accidents qui l'accompagnent. (On the Dentition of Infants and the Disorders attendant upon it:) Inaugural Dissert., Paris, 4to. DUVAL, J. R., Des Accidents de l'extraction des dents. (Of Accidents Resulting from the Extraction of the Teeth:) Paris, pamphlet, 8vo. JOSSE, Analyse de l'émail des dents. (Analysis of the Enamel of the Teeth:) Paris, Journal of Medicine. JUNCKER, Sur les maladies des dents et les maux de tête. (On the Diseases of the Teeth and Painful Affections of the Head:) Braums. LAFORQUE, L., Dix-sept articl. relatifs aux maladies des dents. (Seventeen Articles on Diseases of the Teeth.) The same author, Théorie et pratique de l'Art du Dentiste. (Theory and Practice of the Dental Art:) Paris, 8vo, 1st edition. VAN DER MÆSSEN, Nützliche Belehrung Zur Pflege und Erhaltung der Zahne. (Useful Instruction in relation to the Care and Preservation of the Teeth:) Gotha. MORTET, TOUSS., Dissertation Sur l'extraction des dents a l'aide d'un instruments nouveau. (Dissertation on the Extraction of the Teeth by means of a New Instrument;) An Inaugural Thesis: Paris, 4to.

1803. Bættiger, Charles Aug., Sabina oder Morgenscenen im Putzzimmer einer reicher Römerin. (Sabina, on Morning Scenes in the Dressing-room of a Rich Roman Lady:) Leips., 8vo. Angerman, Translation of Laforgués Theorie et Pratique de l'Art du Dentiste: Gœtt. Aronson, Translation of the above work: Berlin, in 8vo. Van Den Mæssen, (before cited,) Der Zahnartz zun alle Stande. (The Dentist for all Classes of Society:) Leips. Duval, (before cited,) Reflections Sur l'odontalgie Considerée dans ses rapports avec d'autres maladies. (Reflections on Odontalgia Considered in its Relations with other Diseases:) Paris, a pamphlet, 8vo. Fox, Jos., An Account of the Diseases which Affect Children during First Dentition: London. Gallette, Sur l'art dentaire. (Upon the Dental Art:) May. Grousset, De la Dentition on du develop. des dents chez l'homme, et des maladies qui en sont quelqefois le résultat. (On Dentition or the Development of the Human Teeth and the Diseases which sometimes Result from it;) Inaugural Dissertation: 8vo. Rubicki, Dissertatio de Dentitione Difficili: in 8vo, Regio.

1804. Goguelin, J. G., Memoire Sur le Scorbut. (Paper on Scurvy:) Saint-Brieuc, in 8vo. Leroy, Medecine Maternelle. (Maternal Medicine:) Paris, 12mo. Reymondon, Dissert. inaug. Sur la dentition. (Inaugural Dissertation on Dentition.) Rosset, (*idem.*) Jeron, J., Praktesche Darstellung aller Operationen der Zahnartznerkunst. (Practical Exposition of all the Operations of the Dental Art:) Berl. Deschamps, Junior, Traité des maladies des fosses nasales et de leurs sinus. A Treatise on the Diseases of the Nasal Fossæ and their Sinuses:) Paris, 8vo. Rosset, Sur la Dentition. (On Dentition;) Inaugural Dissertation: Paris, 8vo. Zakbockjen Bevattlnde de Middeln om de Gezondheit der tanden te Berwaren, derzelver Zieklyke toevalle te voo rkomen en te Keer to gaan, (Directions for the Preservation of the Health of the Teeth:) J. Arnh.

1805. Bucking, Traité complet sur l'art d'anacher les dents. (Complete Treatise on the Art of Extracting Teeth. Cailleau, (before cited,) Memoire sur la premiere dentition, (On First Dentition:) Bord. Duval, (before cited,) Conseils des Soëtes

Anciens sur la Conservat des dents. (Advice of the Ancient Poets in Relation to the Preservation of the Teeth,) 8vo. Published in the Magasin Encyclop. GARIOT, Traité des Maladies de la bouche, (A Treatise on the Diseases of the Mouth :) Paris, 8vo., with plates. RENGELMANN, CAROL, JOS., De Ossium Morbis eorumque in Specie Dentium Carie: Arnstadt. SCHMIDT, le Moyen de Soigners et mainteuir les dents Saines. (Means of Attending properly to the Teeth and Preserving them in a Healthy Condition.) DUBOIS A., Published in the bulletins of the Society de la Faculté, a remarkable case of dropsy of the maxillary sinus.

1806. BAUMES, Traité de la premiere dentition, et des maladies souvent tres graves qui en dependent. (A Treatise on First Dentition and diseases frequently very serious, which result from it:) Paris, 1 vol. 8vo. DELABARRE, C. F., Dissertation sur l'histoire des dents. (A Dissertation on the History of the Teeth :) in 4to, Paris. FOX, (before cited,) The History and Treatment of the Diseases of the Teeth and Gums, etc. : London, in 4to. GARIOT, (before cited,) Systême de la physiologie, pathologie et therapeutique de la bouche avec des notes d'Angermann. (A system of Dental Physiology, Pathology and Therapeutics, with Angermann's notes :) Leips. SCHMIDT, (before cited,) Theorie et pratique des dents. (Theory and Practice of the Teeth :) Leips.

1807. VAN DER MAESSEN, (before cited,) Comment les parents peuv.-ils faciliter les moyens de faire les dents aux enfants? (How can Parents assist the Operations of Nature in the formation of the Teeth of their Children.) BECKER, Ueber die Zähne und die Sichersten Mittel, etc. (On the Most Certain Means of Curing the Diseases of the Teeth :) Leips., 8vo; There is an edition of 1810. DUVAL, (before cited,) Experiences et observations sur les dents plombées qui sont susceptibles de l'influence galvanique. (Experiments and Observations on those Plugged Teeth which are susceptible of Galvanic Influences:) a pamphlet, 8vo, Paris. The same author, Le Dentiste de la Jeunesse, etc. (On the Diseases of the Teeth of young persons and their treatment:) Paris, in 8vo. JOURDAN and MAGGIOLO,

Manuel de l'art du Dentista. (Manual of the Dental Art:) 8vo, with plates. FOURCROY, Expér sur la nature de l'ivoire frais, fossil, etc. (Experiments on the Nature of Dental Bone recently fossil, etc.:) Memores de l'Institut, etc,, vol. 7. MARTEL, N. M., L'Odontalgie et les malad. qui la simulent. (Tooth-ache, and the diseases resembling it:) Inaugural Thesis, 4to. In a treatise on Accouchement by GARDIEN, published in this year is to be found a very long chapter on dentition and the diseases sometimes occasioned by it.

1808. AUDIBRAN CHAMBLY, Essai sur l'art du dentiste. (An Essay on the Art of the Dentist:) 8vo. The same author, Refutation sur les dents minerales. (Refutation of the alleged advantages of Mineral Teeth:) a pamphlet of 12 pages, Paris. DUBOIS FOUCOU, Exposé des nouv. procédés sour la Confect. des dents detes de Composition. (An explanation of the new process for setting what are known as Composition Teeth:) in 8vo. The same author, Léttre adres à MM. les Dentistes. (A letter addressed to dentists:) Paris, 8vo. DUVAL, (before cited,) Recherch historiq. sur l'art du dentiste (Historical Researches on the Dental Art:) a pamphlet, 8vo, Paris. FONZI, Rapport sur les dents artificielles terro-metalliques. (Report on terro-metallic artificial teeth.) The same author, Réponse à la broch de M. Dubois: Foucon. (Reply to the pamphlet of M. Dubois: Foucon.) HERNANDEZ, Paper on the following questions proposed by the Society of Medicine of Lyons: Quels sont les signes diagn. et prognos que pent fournir dans les maladies aigües et chroniques l'etat de la langue, des lèvres et des dents? Quelle conséquence doit on en déduire dans la pratique? (What diagnostic and prognostic indications are furnished by the tongue, the lips and teeth in acute and chronic diseases? What conclusions should be drawn from them in practice?) a pamplet, 8vo. Toulon. MIEL, Descript. d'un nouveau instrument pur exècutor facilement une operat. occasion. par la fracture des pivots dans les racines, etc. (Description of a new instrument for the easy performance of the operation occasioned by the breaking of pivots in the roots of teeth:) a pamphlet, 8vo, Paris.

1809. Albrecht, Sichere Mittel gegen das Zanweh.) Certain Method of Curing Tooth-ache :) Hamb. Rudolphi, Dissertatio, observat. Circà Dentitionem: Greifs., in 4to. Millot, Médecine Perfective, on Code des bonnes meres, (Improved (?) Medicine on the Code of Good Mothers:) Paris, in 8vo.

1810. Duval, (before cited,) Memoire sur la position relat. de l'ouvert. extér. de canal maxil. pour servir à la demonst. de l'accroissement de la mach. infer. Paper on the Relative Position of the Opening of the Maxillary Canal, to serve as a demonstration of the increase of the inferior jaw. The same author, Propositions sur les fistules dentaires. (Propositions in relation to Dental Fistulas :) Pamphlet, 8vo, Paris. Galette, (before cited,) Reflections sur la cure des dents : May. Laforgue, (before cited,) Theorie et pratiq. de l'art du dentiste. (Theory and Practice of the Dental Art :) new edition, in 2 vols. with plates, Paris. Miel, (before cited,) Note sur la manière dent les dents sortent des alvéoles et traversent les gencives. (Note on the manner in which the Teeth rise from the Sockets and pass through the gums :) a paper read before the Medical Society of Emulation, pamphlet, 8vo.

1811. Murphy, Jos., A Natural History of the Human Teeth, with a Treatise on their Diseases from Infancy to Old Age, etc., 8vo, London. Leveille, J. B. F., Sur les rapports qui exist. entre lres et les 2mes dents, et sur la disposition favorable de ces dernières au developpement des deux machoires. On the Relations which exist between the First and Second Teeth, and on the Favorable Disposition of the last to the Development of the two Jaws :) Memoires de la Soc. méd d'emulation, tome vii. Miel, (before cited,) Quelques idées sur le rapport des deux dentitions et sur l'accroissement des machoires dans l'homme. (Some Ideas on the Relations of the Two Dentitions, and on the Increase of the Human Jaws :) a paper read before the Medical Society of Emulation, and inserted in the same volume as the preceding.)

1812. Auvity, Antoine, Premi re dentition et sevrag; (On First Dentition and Weaning ;) Inaugural Dissertation, 12mo. Paris. Lemaire, Jos., Le Dentiste des dames; (The

Ladies' Dentist;) 12mo. Paris LEGROS, The Conservateur des dents; (The Preserver of the Teeth;) Paris. LICHTENSTEIN, J. M., Ueber die Sorgfult für Zahnfleisch und Zähne;) On the Cares required by the Gums and Teeth;) Brem. 8vo. LAVAGNA, FR,. Esperienze e riflessioni Sopra la carie dé denti humani coll'aggiunta di un nuovo Saggio Su la riproduzione de denti negli animali rossicanti; (Experiments and Reflections on Caries of the Human Teeth, with a supplement on a new subject; the reproduction of the teeth of the rodentia class of animals;) Geneva, 8vo.

1813. GALLETTE, (before cited,) le Chirurg. Dentiste; (The Surgeon Dentist;) A pamphlet, 12. FINOT, C. F., Maladies de la première dentition; (Diseases of First Dentition;) Inaugural Dissert. 4to., Paris. SAUCEROTTE, Avis sur la conservat. des dents; (Advice in relation to the Preservation of the Teeth;) 12mo., Paris. SERRE, Praktische Darstellung aller Operationen der Zahnartzneikunst; (Practical Exposition of all the operations of Dental Surgery;) Berlin. LISFRANC, J., Quelq. propos. de pathologie precédés de recherch., reflex., et observ.; Sur l'amputat. de la mâchoire inferieure, nécesessitée par un fungus dégénéré en cancer, etc.; (Some Propositions on Pathology; preceded by researches, reflections and observations, on the Amputation of the Lower Jaw, rendered necessary by a Fungus Degenerated into Cancer, etc.;) Inaugural Dissertation, 4to., Paris. RIVEIERE, J. L., Instruction pour Conserver les dents belles et saine, aux diverses époques de la vie; (Instructions on the Means of Preserving the Teeth in Fine, Healthy Condition, at the Various Epochs of life;) 12mo. Paris.

1814. DUVAL, (before cited,) Observat. Sur l'état des os de la mach. dans les ulcer. fistul. des genciv., etc.; (Observations of the Condition of the Bones of the Jaws, in Fistulous Ulcers of the Gums;) Bulletins of the Faculty, No. 10. The same Author, Observat. Sur quelq. affect. donleur. de la face, Considerées dans leur rapport avec l'organe dentaire; (Observations on some Painful Affections of the Face, Considered in their relation with the Dental Organs.) LAFORGUE, (before cited,) De la Semeiologie Buccale et buccamancie; 8vo., Paris.

CUVIER, F., The word *Dents.* (tooth) (*Anatomie*;) Fournier—Pescay, The same word, (*Pathologie*;) MURAT, The word *Dentition*, in the large Dictionary of Medical Sciences. TOUCHARD, Description d'un obturateur dentier présenté a la Société de Med. de Paris; (Description of an obturator Set of Teeth, Presented to the Medical Society of Paris;) 8vo.

1815. DELABARRE, (before cited,) Odontalgie ou Observ. Sur les dents humaines Suivies quelques idées Sur le mecanisme des dentiers artificills; (Odontalgia or Observations on the Human Teeth, followed by some ideas on Artificial Sets of Teeth;) Paris, 8vo. TULLER, A Popular Essay on the Structure, Formation and Management of the Teeth; 8vo. London. This work was translated by Downing. HERTZ, J. P., A Familiar Dissertation on the Causes and Treatment of Diseases of the Teeth; London, 8vo.

1816. AUBRY, J. B. L., Maladies des gencives; (Diseases of the Gums;) Inaugural Dissertation, 4to. Paris. LAFORGUE, (before cited,) le Triomphe de la premiere dentition; (The Triumph of First Dentition;) Pamphlet, 32mo. DUVAL, (before cited,) Notice Historiq. Sur la vie et les ouv, de Jourdain; (Historical Notice of the Life and Works of Jourdain;) 8vo. pamphlet. LEMAIRE, (before cited,) deux observ. d'anatomie pathologique Sur les dents; (Two Observations on the Pathological Anatomy of the Teeth;) pamphlet of five pages. RICCI, (before cited,) Memoire Sur les dents raciformes ou racisuberiques; (A Paper on raciform or racisuberic teeth;) 8vo. Paris. The same author, Instruction Sur l'entretien des dents et des gencives, Sur les propriétés d'une liqueur utile pour la guerison de leurs affections, etc.; (Instructions in relation to the Means of Preserving the Teeth and Gums, upon the Properties of a Wash, useful for the Cure of their Diseases, &c.;) pamphlet, 8vo. LEROY DE LA FAUDIGUERE, Manière de prévenir et guérir les maladies des gencives et des dents, précédé d'un avis sur son elixir par sa fille epouse of M. Duval; (Manner of Curing the Diseases of the Gums and Teeth, preceded by a Notice of his Elixir, by his Daughter, wife of M. Duval;) pamphlet, 8vo.

1817. Delabarre, (before cited,) Discours d'ouverture d'un cours de medecine dentaire; (A Lecture, Introductory to a Course of Dental Medicine;) pamphlet, 8vo. Paris. Serres, E. R. A., Essai Sur l'anatomie et la physiologie des dents on Novelle theorie de dentition; (An Essay on the Anatomy and Physiology of the Teeth, or a New Theory of Dentition;) Paris, 8vo.

1818. Cornelio, Vict., Statistica odontalgica del Piemonte, et in especie di Torini per l'anno 1817; (Odontalgic statistics of Piedmont, particularly of the City of Turin, for the year 1817;) Turin. Parmly, L. S., A Practical Guide to the Management of the Teeth, with its prevention and cure; Lond. 12mo. Regnart, Mémoire Sur un nouveau moyen d'obturation des dents; (A paper upon a new Method of Plugging Teeth;) pamphlet, 8vo. This new method is nothing more than the use of D'Arcet's mineral, rendered more fusible.

1819. Bew, Charles, Opinions on the Causes and Effects of Diseases of the Teeth and Gums, etc.; London. Delabarre, (before cited,) Traité de la deuxième dentition et nouvelle méthode de la diriger, etc.; (A Treatise on Second Dentition, a New Method of directing it, &c.;) Paris, 8vo., with plates. Guertin, Avisos tendentes a conservaçao dos dentes et Sua substituiçao; (Advice on the Preservation and Replacement of the Teeth;) Paris, a pamphlet 8vo., of 8 pages. Neumark, Der Zahnartz f r Nichtartze; (The Dentist for the Public;) Berlin.

1820. Manière de conduire les enfants depuis leur naissance, etc.; (Method of Managing Children from the Time of Birth;) pamphlet, 8vo. Delabarre, (before cited,) Traité de la partie méchanique de l'art du chirurgien dentiste; (Treatise on the Mechanical Branch of Dental Surgery;) Paris, 2 vol., 8vo. Duval, (before cited,) De l'arrangement Se des secondes dents ou Méthode nouvelle de diriger la deuxieme dentition, etc.; (On the Arrangement of the Second Teeth, or a new Method of Directing Second Dentition, &c.;) 8vo. Maury, J. G. F., Manuel du Dentiste pour l'application des dents artificielles incorruptibles; (Manual of the Dentist for the Application of

Incorruptible Artificial Teeth;) 8vo. PARMLY, (before cited,) Lectures on the Natural History and Management of the Teeth; Lond. 8vo. ROUSSEAU, EM., Dissertation inaugural Sur la première et la deuxième dentition; (Inaugural Dissertation on First and Second Dentition;) Paris, 4to.

1821. AUDIBRAN, (before cited,) Traité historiq. et pratiq. Sur les dents artificielles incorruptibles; (Historical and Practical Treatise on Incorruptible Artificial Teeth;) 8vo. LEMAIRE, (before cited,) Histoire naturelle des dents de l'espèce humaine trad. de l'anglais de Jos. Fox; (Translation of Fox's Work on the Teeth;) 4to., with plates; Paris. PARMLY, ELEAZ., An Essay on the Disorders and Treatment of the Teeth; London, 8vo.

1822. CUVIER, F., Des dents des mammif res Cansidérées comme caractère zoologique termin en 1825; (The Teeth of the Mammiferous Animals, considered in their zoological character; concluded in 1825;) 8vo., with 100 plates LEMAIRE, (before cited,)Traité Sur les dents; (A Treatise on the Teeth;) 3 vol., 8vo. Concluded in 1824. LUDOLF, H., Disputatio de Morbis Gengivarum; edit du memoire de 1708. FRAENKEL, De l'utilité et des inconvenients des dents artificielles; (Of the Uses and Inconveniences of Artificial Teeth;) Biblio von loeger, tome 2.

1823. OUDER, J. L., Expériences Sur l'accroiss continuet la reproduct. des dents chez les lapins consid. dans leur applicat. à l'étude de l'organis. des dents humanis; (Experiments on the Continued Growth and Reproduction of the teeth of Rabbits, Considered in their Application to the Study of the Organization of the Human Teeth;) pamphlet, 8vo. GERBEAUX, A Practical Treatise on the most frequent Diseases of the Mouth and Teeth, especially on the Accidents of First Dentition; London, 8vo. LEVEQUE, Notice Sur la nécessité de diriger la dentition des enfants, les Soiris que réclam. les dents à tons les âges, et les moyen à employer pour prevénir, arrêtir ou ralenter les progrès des maladies qui affectent les organes; (Notice on the Necessity of Directing the Dentition of Children, the Care claimed by the Teeth at all Ages, the Means to be

Employed to Preserve them, and to Arrest or Palliate the Diseases to which they are Subject;) 8vo., Strasburg. Toriac, Dissert. inaug. Sur les dents Consider es Sous le rapport de la Santé, de la physiognomie, de la pronunciation ; (An Inaugural Dissertation on the Teeth, considered in their relations to the Health, the Physiognomy, and the Pronunciation;) 8vo. Paris. Marjolin, The word *Dent, Pathologie;* (Tooth, Pathology ;) Beclard, the same word, *Anatomie*; (Anatomy ;) Guersant, pére, the word *Dentition*, in the Dictionary of Medicine. Ringelman, Abhandlung über die dicet pflege des Mundes und Erhaltung gesunder reiner Zähne; (Treatise on the Care of the Mouth and the Preservation of the Teeth in a Cleanly and Healthy Condition;) Nürnburg. Dubois, Esquisse Sur l'hygiene dentaire, ou analyse des moyens propres à la Conservation des dents et des gencives; (Sketch of Dental Hygiene or Analysis of the Means proper to the Preservation of the Teeth and Gums;) 8vo, Paris.

1824. Delmond, Mémoire sur un nouveau procédé pour detruire le Cordon dentaire des six dents anterieures et éviter leur extraction; précédé de quellq. reflex. Critiq. Sur l'opinion de M. Lemaire, qui Soutient que les dents Sont des Corps inorg. et nullement Soumis à l'empire de la vie animale. (A Paper on a New Process of Destroying the Pulp of the Six Anterior Teeth, and avoiding the Necessity of their Extraction; preceded by some Critical Reflections on the Opinion of M. Lemaire, who Maintains that the Teeth are Inorganic Bodies and are not in Vital Connection with the Animal Economy:) 8vo, pamphlet. Dubois de Chemant, (before cited,) Dissert. Sur les advantages des dents incorruptibles de paté miner., etc. (A Dissertation on the advantages of the Incorruptible Mineral Teeth:) 8vo, pamphlet. Geoffroy Saint-Hilaire, Système dentaire des mammifères et des oiseaux, etc., embrassent Sous de nouv. rapports les princip. faits de l'organisat. dentaire ches l'homme. (Dental System of the Mammiferes, of birds, &c., bringing together under New Relations the Facts Known of the Human Dental Organization:) pamphlet of 24 pages, Paris. Tronbat, Accidents d'une dentition difficile; moyen certain d'y remédier.

(Disorders Consequent on a Difficult Dentition; Certain Method of Relieving them:) Mayen. 8vo.

1825. DELABARRE, (already cited,) Discours d'ouverture d'un Course de Stomatonomie. (Introductory Lecture to a Course on Stomatonomy:) pamphlet, 8vo. MARMONT, J., L'Odontotechnic, poeme didacteq. et descript. en 4 chants, etc. (Odontotechny, a Didactic and Descriptive Poem in 4 Cantos:) 1 vol. 12mo, Paris. DELMOND, (before cited,) Epître à M. Marmont à l'occasion de Son Poëme Sur l'Odontotechny. (Epistle to M. Marmont on the occasion of his Poem on Odontotechny:) pamphlet, 8vo. MAURY, (before cited,) Traité Complete de l'art du Dentiste. (Complete Treatise on the Dental Art:) 1 vol. 8vo, with plates. There are two other editions of this work, one published in 1833, and the other in 1838. DUVAL, (already cited,) Extrait d'un memoire Sur l'atrophie des dents. (Extract from a Paper on Atrophy of the Teeth.) The same author, Notice des travaux entrepris en France Sur les dents depuis 1790. (Notice of the Works on the Teeth undertaken in France since 1790:) Paris, 8vo. ROUX, PH. JOS., Memoire Sur la Staphyloraphie. (A Paper on Staphyloraphy:) Paris, 8vo, with illustrations. SIGMOND, A Practical and Domestic Treatise on the Diseases and Irregularities of the Teeth and Gums, with the Methods of Treatment: 8vo, Bath. FATTORI, De la trépanation des Dents. (On Trepanning Teeth:) See the *Revue Medicale*, number for February. TALMA, Notice Sur quelq. erreurs relatives à l'art du dentiste et Sur l'emploi de la lime et de la cautérisat. dans la carie Superfi. (Notice of some errors Relative to the Dental Art upon the use of the File and Cauterization in Simple Caries:) Brussels, 8vo. VAUQUELIN, Rapport Sur le tartre des dents, fait à la Section de pharmacie, de l'Academie roy. de Medecine. (Report on Dental Tartar made to the Section on Pharmacy of the Royal Academy of Medecine.)

1826. DEVANT, Essai Sur la nature et la format. des dents. (Essay on the Nature and Formation of the Teeth:) Inaugural Dissertation, 4to, Paris. CATALAN, Memoire, rappart et observations Sur l'appareil propre à corriq. la difformité vulgarien. nommé menton de galoche. (Report and Observations on the

Apparatus Proper for the Correction of the Deformity of a Long Pointed Chin.) OUDET, (before cited,) Considerat. Sur la nature des dents et leurs maladies. (Considerations on the Nature of the Teeth and their Diseases.) Journal univers. des Sc. m dic., tome iii. DELABARRE, (before cited,) Methode Naturelle de diriger la deuxième dentition. (Natural Method of Directing Second Dentition:) Paris, 1 vol. 8vo, with illustrations. KŒCKER, LEONARD, Principles of Dental Surgery, exhibiting a New Method of Treating the Diseases of the Teeth and Gums: London, 8vo. MIEL, (before cited,) Recherches Sur l'art de dirigir la deuxieme dentition en général. (Researches on the Art of Directing Second Dentition in General.)

1827. FAY, A Description of the Mode of Using the Forceps, invented for the extraction of Teeth: a pamphlet, 8vo, London. GOBLIN, D., Manuel du dentiste, à l'usage des examens. (Manual of the Dentist for the Use of Examination:) 8vo, Paris. ROUSSEAU, (before cited,) Anatomy Comparée du système dent. Chez l'hom. et les princip. anim. (Comparative Anatomy of the Dental System in Man and the Principal Animals:) 8vo, with plates. TAVEAU, O., Hygiene de la bouche, on Traité des soins qu'exig. l'entret. de la bouch. et la Conserv. des dents. (Hygiene of the Mouth, or a Treatise on the Care necessary for the Preservation of the Health of the Mouth and Teeth:) 1 vol. 12mo, Paris. There are a number of editions of this work; the last was published in 1843, in octavo.

1828. AUSSANT, Dissert. inaug. Sur les Soins à donner aux dents de deuxieme dentition. (Inaugural Dissertation on the Cares required by the Second Teeth:) 4to. TAVEAU, Conseils aux fumeurs Sur la Conservation de leurs dents. (Advice to Smokers in relation to the Preservation of their Teeth:) 8vo, Paris.

1829. TOIRAC, (before cited,) Memoire Sur les diverses espèces de déviations dont est susceptible la dernière dent molaire. (A Paper on the various kinds of Deviations to which the last molar tooth is Susceptible:) 8vo, Paris. BOUCHARD, Maladies que determine la première dentition. (Diseases occasioned by First Dentition:) Inaugural Dissertation.

1830. VIDAL, A., De Morbis Maxillaris Inferioris, etc., (Inaugural Thesis.) LESDAY, De Dentitione Primâ et Secundâ Investigationibus Novis Illustratâ: Vindson.

1831. BEGIN, Article *Dent* (Tooth) in the Dictionary of Practical Medicine and Surgery. DUGES, A., Article *Dentition*, on the same Dictionary.

1832. DEPLOEG, D. E., Le Dentiste populaire, on l'Art de Soigner et d'entretenir les dents, Suivi de la description d'un nouveau perforateur pour les racines. (The Popular Dentist or the Art of Preserving the Teeth, followed by a description of a New Perforator of the Roots:) a pamphlet, 8vo, Brussels.

1836. BLANDIN, PH. F., Anatomy du Système dentaire Considéré dans l'homme et les animaux. (Human and Comparative Anatomy of the Dental System;) These de Concours: Paris, 1 vol. 8vo, with plates. DONNE, ALPB., Historie physiologiq. et pathologiq. de la Saline, Considerée particulierement Sous le rapport de ses usages. (Physiological and Pathological History of the Saliva, considered particularly in Relation to its Uses:) pamphlet, 8vo, Paris. OUDET, (before cited,) The word *Dent* (Tooth) of the Dictionary or General Repertory of the Medical Sciences, vol. 30.

1837. TAVEAU, O., (before cited,) Notice Sur un Ciment oblitérique pour arretir et guérir la carie des dents. (Notice of a Cement for the purpose of Arresting and Curing Caries of the Teeth:) pamphlet, 8vo, Paris. PATTERSON CLARK, J. A., Practical and familar Treatise on the Teeth.

1838. DESIRABODE, ED., Considerations anatomiq., physiologiq. et pathologiq. Sur les Dents de Sagesse. (Anatomical, Physiological and Pathological Considerations in relation to the Wisdom Teeth:) Inaugural Dissert.

1839. RASPAIL, Recherches sur la cause première et la médication de la carie dentaire. (Researches on the first cause and treatment of Dental Caries:) Gazette des Hopitaux, Number for January 22d. DUVAL, (before cited,) Considerations sur l'ivoire, etc., (On the Dental Bone:) the same Journal number for January 26th. LEFOULON J., Orthopedie dentaire (Dental Orthopedia:) see the same Journal, numbers for 5th, 6th and 9th of March.

1840. Regnart, (before cited,) De la Carie dentaire. (Dental Caries:) pamphlet, 8vo. Serrurier, Réfutation de la brochure de M. Regnart sur la carie. (Refutation of M. Regnart's pamphlet on Caries:) Gazette des Hopitaux, for September 20th, 25th, and December 11th. Brewster, Developpem anornal de la portion antér du maxil. sup. avec rétractation de la lèvre supérieure. (Abnormal Development of the Anterior Portion of the Superior Maxillary Bone, with Retraction of the Upper Lip:) same Journal, No. for 14th Nov. In the No. for June of the General Archives of Medicine, is recorded a very curious case of late dentition. Forget, A., Des kystes qui se developpent dans les os maxillaries. (Of the Cysts Developed in the Maxillary Bones:) Inaugural Thesis, 8vo.

1841. Lefoulon, (before cited,) Nouveau traité theorique et pratique de l'art du dentiste. (New Theoretical and Practical Treatise on the Dental Art:) Paris, 1 vol., 8vo.

1842. Schange, J. M. A., Precis sur le redressement des dents, suivi de quelques reflex. sur les obturateurs palatins. (Compend on the Correction of Irregularities of the Teeth, followed by some reflections on Palatine Obturators:) 8vo, Paris. Talma, Memoire sur les douleurs Dentaires qu'on peut confondre avec certaines neuralgies faciales: présenté a l'Académie royale de Med. (A paper on the Dental Pains, which may be confounded with certain Neuralgic Affections of the Face:) See the Bulletins. M. Nasmyth, of the College of Surgeons, London, read on the 5th of December, at the Institute, a paper on the Organization of the Teeth. See the records of the meeting, and also what we have said on p. 221.

1843. Billard, Memoire sur les dents minerales. (A paper on Mineral Teeth,) a pamphlet, 8vo. In the No. for the 4th of February of the Gazette des Hôpitaux, we find a very curious article from Dr. Huguier, on Alveolar Exostosis of the Maxillary Bones. Mandl, Recherch. microscopiq sur le tartre, etc. (Microscopic Examinations of Tartar:) read before the Institute, at the sitting of July 28th. The object of these examinations was to prove that the tartar is nothing else but an accumulation of the skeletons of small insects which form around the teeth; this is but a variation of the opinions of Magellan and Leuwenhoeck, who wrote about the middle of the seventeenth century. In the sitting of the Institute of the 21st of August, M. Dumeril, in his name and those of MM. Serres, and Flourens, made a very favorable report on a paper from M. Duvernoy, who attempted to prove that the dental pulp is the producing organ of the dental bone, and that the enamel is deposited in successive layers around the crown by a membrane distinct from the capsule. Desirabode, père et Fils Nouveaux elements complets de la science et de l'art du dentiste: Paris, 2 vol., large 8vo, avec une notice historique et chronologique des principaux ouvrages publiés sur l'art du dentiste depuis Hippocrate jusq'ā nos jours. (New Complete Elements of the Science and Art of the Dentist; with a Historical and Chronological notice of the principal works published on the art, from Hippocrates down to the present time.

